# Base-Rate Error in the Interpretation of Immunohistochemistry

**DOI:** 10.4061/2011/636495

**Published:** 2011-05-26

**Authors:** Malcolm Galloway

**Affiliations:** Department of Cellular Pathology, Royal Free Hospital, Royal Free Hampstead NHS Trust, Pond Street, Hampstead, London, NW3 2QG, UK

## Abstract

Failure to appreciate the importance of the frequency of a disorder in the appropriate population (the base rate) may lead to the misinterpretation of the diagnostic significance of unexpected test results (unexpected test result defined in this context as a test result that is positive in a higher proportion of cases of an alternative diagnosis than in the diagnosis considered most likely before the test). This study aimed to determine whether pathologists are vulnerable to this error. Pathologists were asked to estimate the probability of tumour B in a scenario in which, prior to the immunostaining result, an experienced pathologist considers there to be a 99% chance that the patient has tumour A and a 1% chance that they have tumour B. Antibody X is positive in 80% of cases of tumour B and negative in 90% of cases of tumour A and is positive in the case described in the scenario. The estimates made by consultant pathologists ranged from 0 to 100% (mean 29.7%). The Bayesian answer would be 7.5%. These findings suggest that base-rate error may lead some pathologists to overestimate the implications for the likelihood of a diagnosis in the light of an unexpected immunohistochemical result.

## 1. Introduction

Base-rate error in medical diagnosis refers to the cognitive bias in which doctors may underestimate the importance of the frequency of the relevant disorder in the appropriate population (the pretest probability) when considering the implications for the diagnosis of the result of tests which have less than 100% sensitivity and specificity. An underappreciation of the importance of the base rate has been shown outside medical practice, for example, in the interpretation of fallible witness evidence [1], and has also been shown in relation to the interpretation of diagnostic test results by physicians [1–3].

In the field of base-rate errors in diagnostic interpretation, the most closely studied area thus far has been in relation to mammography. Eddy has previously reported base-rate error in the interpretation of the diagnostic contribution of mammogram results by physicians [3]. In his scenario, 79.2% of patients with breast cancer are assumed to have a malignant result on mammography (sensitivity) and 90.4% of patients without cancer to have a benign mammogram result (specificity). He asked what he described as “an informal group of approximately 100 physicians” what the probability of a patient having breast cancer would be if they had a 1% risk of the disease prior to the mammogram but had a malignant diagnosis on mammography. In this scenario, the mathematically correct answer (by Bayesian analysis) would by 7.7%. He reported that approximately 95% of physicians estimated the risk as approximately 75% (almost 10 times the actual risk). 

A similar study in which physicians were asked to predict the probability of a malignant lesion in a patient with a positive mammogram result demonstrated that even when providing their own individual estimates for the sensitivity and specificity of mammography in a breast mass scenario, physicians consistently overestimated the impact of a positive test result on the likelihood of the disease [4]. 

Pathologists are regularly formulating differential diagnoses and using further investigations, particularly immunohistochemistry, to assist in the selection of the final diagnosis. This process could be regarded as cognitively equivalent to the processes involved in making a clinical differential diagnosis of a breast mass, further investigating with mammography, and then deciding whether to proceed to biopsy. As with mammography, immunohistochemistry will rarely if ever be 100% sensitive and specific in the differentiation between two or more entities but may assist in making a diagnosis more or less likely, in the context of the prior probabilities of the diagnosis as assessed by the pathologist on morphological grounds, in the context of the clinical and radiological background data. This is not to suggest that a pathologist in real-life practice would ever consciously undertake a Bayesian analysis on the immunocytochemical results of a test; however, I would argue that the possibility that immunocytochemical testing might be subject to base-rate-related cognitive error is sufficiently plausible to warrant investigation. If such an error were to be widespread, this would suggest that pathologists might be at best at risk of unnecessary expense in ordering investigations that minimally affect the probability of the differential diagnoses, and at worst might lead to misdiagnosis and inappropriate treatment. To the best of my knowledge the risk of base-rate error specifically in relation to the use of immunohistochemistry has not been previously investigated.

The study reported here used a scenario mathematically similar to that used in Eddy's mammography study [2], however the content and context was altered to reflect the practice of histopathology.

## 2. Materials and Methods

A survey was conducted of cellular pathology consultants and trainees in which the following scenario was presented:

“Please read the following hypothetical scenario: An experienced histopathologist after examining a haematoxylin and eosin stained slide and taking into account the clinical and radiological context of the patient considers there to be a 99% chance that the patient has tumour A and a 1% chance that they have tumour B These tumours require different treatment. Antibody X has been shown to be positive in 80% of cases of tumour B, and negative in 90% of cases of tumour A. The pathologist is surprised to find that the lesion stains convincingly for antibody X. What would you estimate are the chances that the patient has tumour B? (Please state estimated percentage)”.

By Bayesian analysis, the “correct” answer can be derived from the following equation:

(1) $$\begin{array}{c} P\left(\text{B}\;|\;\text{pos}\right)=\frac{P\left(\text{pos}\;|\;\text{B}\right)P\left(\text{B}\right)}{\left(P\left(\text{pos}\;|\;\text{B}\right)P\left(\text{B}\right)+P\left(\text{pos}\;|\;\text{A}\right)P\left(\text{A}\right)\right)}. \end{array}$$

In the above equation, *P*(B | pos) refers to the probability that the patient has tumour B given the positive stain for antibody X, *P*(pos | B) refers to the probability that if the tumour is B then it will stain for antibody X (sensitivity), *P*(B) refers to the prior probability that the patient has tumour B, *P*  (pos | A) refers to the probability that if the tumour is A then it will stain for antibody X (false positive rate), and  *P*(A) refers to the prior probability that the patient has tumour A.

In the example of this scenario, the figures would be: *P*(B | pos) = 0.8 × 0.01/((0.8 × 0.01)+(0.1 × 0.99)) = 0.075  (7.5%). 

The intention of the study was not to see whether the subjects could perform the calculation but to see how closely their intuitive estimate matched the mathematical calculation.

The question was part of an anonymous survey related to postgraduate pathology education, which had the National Health Service research ethics committee and Royal Free Hampstead NHS Trust Research and Development Department approval. Four groups were surveyed—consultant cellular pathologists (cellular pathologists here defined as histopathologists, cytopathologists, and neuropathologists), trainee cellular pathologists, medical doctors (consultants and trainees) who are not cellular pathologists, and medical students (4th year medical students at the University College London Medical School). All doctors surveyed were practicing in the United Kingdom at the time of the study.

The survey was conducted predominantly online (with invitations to undertake the survey included within the email newsletter of the Royal College of Pathologists and also sent by email to personal contacts in the UK). A minority of responses, predominantly those provided by medical students before lectures and tutorials, were obtained in paper form, and some of these forms distributed to medical students were restricted to the question related to base-rate error.

As shown in Table 1, the numbers of responses were 82 (43), 38 (28), 23 (16), and 59 for consultant pathologists, trainee pathologists, other doctors, and medical students, respectively (with the numbers in parenthesis referring to the number responding to the specific question in the survey addressed in this paper). Many of the medical students were only given the question on base-rate error; therefore, the response rate for this question in the medical student group cannot be assessed.

It is acknowledged that this method of sampling has limitations, including potential sample bias and response bias, and also that it is impossible to reliably assess the response rate to the survey; however, according to the Royal College of Pathologists workforce department, in 2007, there were 1448 consultant cellular pathologists and 555 trainees in the United Kingdom (personal communication). Therefore, it can be estimated that responses were obtained from approximately 3.0% of the consultant cellular pathologists and 5.4% of the trainee pathologists practicing in the United Kingdom.

## 3. Results and Discussion

**Figure 1:**
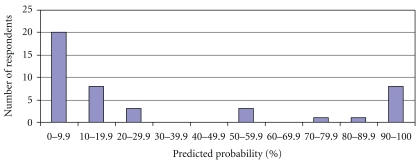
Distribution of predicted probability of tumour B—consultant cellular pathologists.

All four groups showed remarkably similar overall results (see Table 1 and Figure 1). There was an extremely wide range of individual estimates in all groups. Each group contained individual responses giving estimates of the probability of tumour B of under 1% (including one response of 0% by a consultant cellular pathologist and responses of 0.08% by trainee pathologists and a medical student). The upper range of the estimates was also very high in all groups, including three responses of 99% and one of 100% in the consultant cellular pathologist group and up to 99% in the trainee group.

There were no statistically significant differences between the mean estimate of probability between the four groups, as assessed with either independent two-tailed *t*-tests or one-way ANOVA assessments (significance defined as *P* < .05). 

Despite the wide spread of estimates within each group, all four groups produced an identical median estimate probability of 10%. It is of interest that the median estimate of these groups is close to the Bayesian result, whereas the mean results are approximately four times higher. There is no evidence of correlation between experience in making diagnoses (consultants compared with trainees and medical students) and the accuracy of their estimates.

 The distribution of responses within the groups was distinctly non-Gaussian. Figure 1 shows the distribution of responses within the group of consultant pathologists. There appears to be a bimodal distribution, with most of the respondents towards the left of the chart (estimated probability of 0–29.9%), but with a second peak in the 90–100% category. The distribution also failed to follow a Gaussian pattern in the other groups (data not shown). 

The question itself, whether due to the presentation or the content, appeared to be off-putting to some respondents, and it is acknowledged that the explicit description of the probability of a diagnosis is rare in routine diagnostic practice. Only 52% of the consultant pathologists, 74% of the trainee pathologists, and 70% of the nonpathologist medical doctors who undertook the survey provided an answer for the particular question described in this report.

## 4. Conclusion

Despite the limitations of this study, the data would suggest that base-rate error is a potential source of diagnostic error for cellular pathologists. When deciding whether to order and how to interpret the results of an immunocytochemical test, it is very rare to have clear data on the pretest probabilities of a disease in the relevant population and the rate and pattern of positivity in the diseases relevant to the differential diagnosis. The published literature is often contradictory regarding the rates of positivity for stains in different tumours, with subjective factors (including personal thresholds for the interpretation of positivity) and local factors (e.g., variations in fixation, pre-treatment, or antibody clones used) making extrapolation from published literature even more challenging. Inevitably, the interpretation of the diagnostic significance of the test results depends on the subjective impression of the pathologist in the context of the clinical information and morphological findings. 

The data presented here demonstrate that, at least in the admittedly artificial setting of a text-based case scenario, consultant and trainee pathologists show enormous variation in their interpretation of the diagnostic significance of an unexpected immunohistochemical result. I am using the term “diagnostic significance” in this context to refer to the weight placed by the pathologist on the finding in the determination of their diagnosis and “unexpected result” in this context to refer to a test result that is positive in a higher proportion of cases of an alternative diagnosis than in the diagnosis considered most likely before the test was performed. 

Previous data regarding the interpretation of unexpected test results in other contexts [1–4] has demonstrated a tendency of doctors to overinterpret the diagnostic significance of the unexpected result. This was demonstrated by some of the respondents to this survey. For example, 38% of the consultant cellular pathologists gave an estimated probability of double or more the mathematically correct answer, and 30% of consultant pathologists and 32% of trainees gave a response of five times or more higher. If this can be extrapolated to routine diagnostic practice, this would suggest that approximately one third of cellular pathologists substantially overestimate the diagnostic significance of unexpected immunohistochemical staining results.

In contrast, 33% of consultant cellular pathologists and 39% of the trainees gave an estimated probability of half or less of the mathematically correct figure. Again, if this can be extrapolated to routine practice, this would suggest that approximately one third of pathologists underestimate the diagnostic significance of an unexpected immunohistochemical result. The distribution range of the answers, with clusters of inappropriately high estimates, would suggest that there may be common cognitive mistakes made in the assessment of disease probability by all groups surveyed.

Although the finding that approximately one third of pathologists underestimated the diagnostic significance of the result, one third overestimated, and another third were reasonably close might not appear to be overly concerning, the range of individual estimates could be considered to be. The finding that positive staining with an antibody more frequently found in tumour B than tumour A altered the estimated probability of tumour B from 1% to between 0 and 100% by different individual consultant cellular pathologists may be of concern.

Gigerenzer et al. has extensively examined how altering the presentation of probability data in both professional and general public settings can improve the interpretation of statistical information [5, 6]. He consistently found that predictions were improved by presenting information as “natural frequencies” rather than percentage probabilities. For example, in the context of this study, the data could have been presented in a natural frequency format as “an experienced histopathologist after examining a haematoxylin- and eosin-stained slide and taking into account the clinical and radiological context of the patient considers that, out of 1000 patients with a similar slide, 990 will have tumour A and 10 will have tumour B. Antibody X is positive in 80% of cases of tumour B; therefore, it is likely that, of these 10 patients with tumour B, 8 will stain positively. On the other hand, of the 990 patients with tumour A, 99 would be expected to stain for antibody X. If the tumour stains for antibody X, how likely is it that the patient will have tumour B?”. Such a presentation was not used in this study, but, in the light of Gigerenzer et al.'s findings in similar scenarios presented to doctors, it is likely that such a presentation would have improved understanding. 

It could be argued that presenting the information in this study in the way that was chosen could have been designed to be misleading, however pathologists rarely have access to information regarding the stains they routinely use that is presented in terms of natural frequencies. Natural frequency presentation takes away some of the cognitive load in such a calculation, by presenting data that is not reliant on the base rate of the condition in the relevant population. Unfortunately, in routine practice, it is at best extremely rare to have a clear indication of the probability of a disease in the relevant population. In the example given, the base rate of relevance is the population of patients who the pathologist would consider to have a similar haematoxylin- and eosin-stained slide. This will to some extent depend on the frequency of the differential diagnosis in the pathologist's referral population, but will also be dependent on the pathologist's own experience, ability, and biases. It is however vital for pathologists when interpreting studies describing the alleged sensitivity and specificity of stains to consider whether the population assessed is relevant to their own practice. For example, a study comparing the value of a stain in discriminating between a certain tumour and normal tissue may suggest greater sensitivity and specificity than a study comparing its value in discriminating between two morphologically similar but clinically distinct tumours.

It appears from the findings in this study that pathologists are not all immune from base-rate errors and that increasing diagnostic experience does not reduce the risk of such errors. The importance of the base-rate probability of a disease on the interpretation of an unexpected immunohistochemical finding is underappreciated by many pathologists. This is a source of potential diagnostic error, and it is suggested that the trainees are made aware of base-rate errors in their training programmes.

## Figures and Tables

**Table 1:** Summary of results.

	Consultant pathologists	Trainee pathologists	Other doctors	Medical students
Number responding to survey	82	38	23	N/A
Number answering question	43	28	16	59
Median predicted probability of tumour B (%)	10	10	10	10
Mean predicted probability of tumour B (%)	29.7	28	28.8	32.3
Range (%)	0–100	0.008–99	1–90	0.08–90.1
Standard deviation	37.1	35.6	28	34.9
